# Lipid- and Multivariate-Based Analyses to Determine Cell Response to pH Variations and Buffer Composition

**DOI:** 10.1007/s10126-025-10421-4

**Published:** 2025-02-15

**Authors:** Ricardo F. S. Pereira, Carla C. C. R. de Carvalho

**Affiliations:** 1 iBB-Institute for Bioengineering and Biosciences, Department of Bioengineering, Instituto Superior Técnico, Universidade de Lisboa, Av. Rovisco Pais, 1049-001 Lisbon, Portugal; 2 Associate Laboratory i4HB-Institute for Health and Bioeconomy, Instituto Superior Técnico, Universidade de Lisboa, Av. Rovisco Pais, 1049-001 Lisbon, Portugal

**Keywords:** pH, Marine medium, Phosphate buffer, Biological buffer, Prodigiosin, Multivariate statistical analysis

## Abstract

During marine bioprocess development, pH control is of paramount importance. In shake flasks, aerobic fermentations usually have no pH control except from the buffering effect provided by buffers in the medium. However, the use of traditional buffers, such as phosphate buffer, can lead to the precipitation of medium components. Good’s buffers may be a sound alternative. Using *Serratia rubidaea* cells and their production of prodigiosin as model system, several Good’s buffers were tested and compared to phosphate buffer. Lipidomics analysis in conjugation with statistical multivariate analysis was performed to determine the cellular response to pH variations and buffer composition. Biomass productivity was similar when using the different buffers, but prodigiosin production was highly influenced and was highest with MES buffer at pH 5.5, reaching 249.8 mg/L, which corresponds to 43.7 mg/g_biomass_. At pH 7.0, the best results were achieved with EPPS, HEPES and TRIS buffer, being these good substitutes for phosphate buffer in marine medium. The results also show that cells adapted the fatty acid composition of their membranes as response to the buffering species present in the growth medium. This is a clear indication that the buffer composition should not be disregarded when developing a bioprocess.

## Introduction

In the marine environment, natural geochemical cycles and the microbiome response to them tend to create localized proton concentration gradients that influence growth and survival of microbes (Lund et al. 2020). The pH is closely linked to all elements of cellular homeostasis: from enzyme conformational stability and kinetics, membrane transport and homeoviscous adaptation to the formation of free radical species (Venn et al. 2013; Ferreira et al. 2015; Ernst et al. 2016; Wang et al. 2019). Since many biochemical reactions are sensitive to small pH variations, close monitoring of this parameter within a biological process is essential but it is usually overlooked during bioprocess development.

Aerobic fermentations are commonly performed in shake flasks without pH control. Therefore, numerous substances with a buffering effect, which balance the hydrogen ions in solution by reversible protonation, have been tested in growth media (Ferguson et al. 1980). Phosphate buffer is known to inhibit the activity of several enzymes, e.g. carboxy-peptidases, fumarases, ureases, kinases, dehydrogenases and enzymes with phosphate esters as substrates, most likely as the result of the phosphate ion functioning as a competitive inhibitor, or to the formation of complexes with polyvalent cations, such as calcium (Ca^2+^) and magnesium (Mg^2+^), which are physiologically indispensable to practically all living organisms (Ugwu and Apte 2004; Ferreira et al. 2015; Xie and Yang 2016). In multinutrient solutions, such as seawater, phosphate solubility depends on the ionic strength and cation composition since Ca^2+^ and Mg^2+^ strongly associate to phosphate anions (Atlas et al. 1976). Media such as marine broth (MB), designed to mimic sea water, have Ca^2+^ and Mg^2+^, and small changes in alkalinity may cause their precipitation (Irving 1926). Both calcium and magnesium phosphates precipitate easily, limiting Ca^2+^ and Mg^2+^ availability in the growth medium, thus affecting several metal ion-dependent biochemical reactions (Ferguson et al. 1980). Phosphorous is an essential nutrient for marine organisms, and it has been predicted to be the limiting nutrient regulating total ocean productivity (Tyrrell 1999).

To understand the effects associated to the limitations caused by marine media buffering on marine bacteria, the present study used a marine *Serratia rubidaea* strain, which produces prodigiosin, as model system to study phosphate buffer alternatives. The strain was isolated from a sample collected at a shallow-water hydrothermal vent in the Atlantic Ocean (Pereira et al. 2023), and presents high adaptability to environmental changes and to media composition (Pereira and de Carvalho 2024a,b). It is a good model bacterial strain to study pH variations since it is able to grow and produce prodigiosin at least between pH 6.2 and 8.6 (Pereira et al. 2023). Prodigiosin, the red pigment produced mainly by *Serratia* spp., has known antimicrobial and anticancer properties (Darshan and Manonmani 2015), but its application has been hampered by the lack of sound bioprocesses for its production at industrial scale.

In the present study, the assays were carried out at different pH values using different buffer species but also at pH 7.0, which is the pH value allowing the production of the highest amount of *S. rubidaea* biomass (Pereira et al. 2023). It is also the intracellular pH in which most biochemical reactions occur for a myriad of microorganisms, since most microbes are neutrophiles, but the chemical nature of the buffer selected may influence their metabolism.

To validate the conditions that induced the highest production of prodigiosin in shake flask, a 2-L bioreactor was used in this study, to allow pH control, and to determine the influence of the buffer species used. Biomass and prodigiosin concentrations were determined after 24 h of cultivation. To assess cellular adaptations at the membrane level, the fatty acid (FA) composition of the cells was determined. Bacterial cells are able to modulate the FA composition of the phospholipids of their cellular envelop as response to both nutrients used in the culture medium and environmental conditions, including changes in temperature, pH and osmotic pressure (de Carvalho 2012; de Carvalho and Caramujo 2018). Changes is fluidity are achieved mainly by modifications in FA chain length, and in the number and position of double bonds in the FA carbon chain. This mechanism, called “homeoviscous adaptation”, is used by cells to maintain membrane fluidity (Ernst et al. 2016), and it is particularly important in extreme and in rapidly changing environments (de Carvalho 2012; Pereira and de Carvalho 2024a). We have previously shown that the *S. rubidaea* strain under study is able to use numerous carbon and nitrogen sources, while adapting the lipid composition of the cellular membrane to the several induced changes (Pereira and de Carvalho 2024a). In the present study, statistical multivariate analyses were carried out to assess the cellular adaptations at the membrane level when phosphate buffer and Good’s buffers (Good et al. 1966) at different pH values are used for cell growth and prodigiosin production.

## Materials and Methods

### Effect of Phosphate Buffer on Medium Without Cells

Phosphate buffer containing Na_2_HPO_4_/KH_2_PO_4_, at 45 mM and pH values between 5.5 and 8.0, was used to prepare the culture media. The optical density of each medium was measured at 600 nm before and after sterilization to assess the precipitation of compounds.

### Bacterial Strain and Growth Conditions

The marine *S. rubidaea* strain was maintained in cryotubes at − 80 °C in marine broth (MB) with 20% glycerol. To prepare cultures, the cells were grown on marine agar plates at 30 °C. After 24 h, one of the red colonies, indicating prodigiosin accumulation, was transferred to a 100-mL Erlenmeyer flask containing 40 mL of MB. The complex medium contained the following compounds: 42.0 g/L of MB (Condalab, Torrejón de Ardoz, Madrid, Spain), 5 g/L of sodium glutamate, 5 g/L of meat peptone (both from Sigma-Aldrich, St. Louis, MO, USA) and 0.2 g/L of iron (III) sulfate (Riedel-deHaën AG, Seelze, Germany), as indicated in Pereira and de Carvalho (2024a, b). The cells were grown for 24 h, in an orbital incubator (Agitorb 200, Aralab, Rio de Mouro, Portugal), at 180 rpm and 30 °C.

### Effect of pH

The influence of the pH on both biomass and product yields was assessed by using buffers at 45 mM between pH 5.5 and 9.0 (Table 1). Minor adjustments to the pH value were done using 12 M hydrochloric acid (Fisher Scientific UK Limited, Loughborough, Leics, UK) or 2 M sodium carbonate (Merck, Darmstadt, Germany). All buffers were either added to the medium and autoclaved, or every component was autoclaved individually and mixed under sterile conditions. All experiments were carried out at least in duplicate.

**Table 1:** Identification of the different buffers used in the medium to grow the marine *S. rubidaea* strain, and respective pH value(s) used. * at 25 °C, Good et al., 19,966; Dawson et al. 1989. ** at 30 °C, this study

Name	Abbreviation	pH range*	pH tested**	Supplier
Phosphate	PO_4_	5.7–8.0	5.5–7.5	Merck, Darmstadt, Germany
3-(Cyclohexylamino)−2-hydroxy-1-propanesulfonic acid	CAPSO	8.9–10.3	8.5–9.0	Sigma, St. Louis, MO, USA
*N*-(2-Hydroxyethyl)piperazine-*N*′-(2-ethanesulfonic acid)	HEPES	6.8–8.2	6.5–7.0	
2-(*N*-Morpholino)ethanesulfonic acid	MES	5.5–6.7	5.5–7.0	
3-Morpholino-2-hydroxypropanesulfonic acid	MOPSO	6.2–7.6	7.0	
Piperazine-*N*,*N*′-bis(2-ethanesulfonic acid)	PIPES	6.1–7.5	7.0	
*N*-(2-Hydroxyethyl)piperazine-*N*′-(3-propanesulfonic acid)	EPPS	7.3–8.7	7.0–8.5	Acros Organics, Geel, Belgium
Tris(hydroxymethyl)aminomethane	TRIS	7.2–9.0	7.0	Eurobio Scientific, Les Ulis, France

### Bacterial Growth in 2-L Bioreactors

To confirm the high productivity in prodigiosin when MES was used, biomass and prodigiosin production was determined in 24-h batch cultures in fermenter. Two 2-L Fermac 360 bioreactors (Electrolab Biotech, Gloucestershire, UK), with 1.5 L of working volume, were used. Batch cultures were performed at 30 °C and at 40% saturation of dissolved oxygen (DO). A DO control cascade was used, where stirring was kept between 450 and 600 rpm and air flow between 0 and 1 vvm. The pH was controlled automatically either by the addition of 2N NaOH (Panreac, Germany) or 3N H_2_SO_4_ (Sigma-Aldrich, USA). Control cultures without MES contained only MB + S and the pH was controlled by acid and base additions as described.

### Analytical Methods

Dry cell weight (DCW) values of *S. rubidaea* cells were obtained by converting optical density (OD) measurements made at 600 nm in 96-well microtiter plates, using a microplate spectrophotometer (Multiscan Go from Thermo Fisher Scientific, MA, USA). An appropriate calibration curve of DCW vs OD was made, which was checked periodically by measuring the OD of samples and weighting the respective cell pellets dried at 65 °C after 24 h. The OD of the samples was always measured against a blank containing the respective medium without cells. In media containing suspended precipitates, the flasks were manually shaken prior to sampling to obtain homogeneous samples. The microtiter plates were also shaken inside the spectrophotometer prior to OD measurement.

The prodigiosin concentration was determined after cell disruption. For this, 1 mL of sample was taken from the culture medium and *S. rubidaea* cells were harvested by centrifugation at 10,000 × *g* for 5 min, and washed with milli-Q water. The cell pellet was disrupted using acidified ethyl acetate (25% acetic acid) and left for 1 h at room temperature covered from light. After disruption, the cells suspension was centrifuged at 7,000 × *g* for 10 min at 4 °C. Prodigiosin concentration was determined by absorbance measurements at 535 nm in a double beam spectrophotometer (T80 from PG Instruments), using the Lambert–Beer law with an extinction coefficient of 32.2 L mg^−1^ cm^−1^ (Cox and Charles 1973; Mancini and Imlay 2015). To assess changes in the FA composition of the cellular membranes, the lipid content of the cells was extracted, and the FA composition determined by gas chromatography, using a gas chromatograph with a flame ionization detector (GC-FID), as previously described by Pereira and de Carvalho (2024a, b). The identification of the peaks was confirmed by injecting the samples on an Agilent 7820A gas chromatograph, with a 25-m-long Agilent J&W Ultra 2 capillary column, and equipped with a 7693A autoinjector and a 5977E quadrupole mass spectrometer (MS; Agilent Technologies, Santa Clara, CA, USA). Helium was used as the carrier gas at 1.5 mL/min.

### Multivariate Analysis

The Principal Component Analysis and Partial Least Squares (PLS) models were calculated using Metaboanalyst 5.0 (Pang et al. 2021) and Minitab^®^ Release 14.1 from Minitab, LLC (State College, PA, USA) statistical software. The data initially was standardized, for both PCA and PLS analysis, by subtracting the corresponding mean and dividing by the standard deviation.

## Results and Discussion

The *S. rubidaea* strain used in the present study was isolated from a sample collected near a shallow-water hydrothermal vent at Ferraria thermal springs in S. Miguel island, Portugal (Pereira et al. 2023). The sample collected was at 28 °C and pH 8.91. The pH value of the water resulting from the mixture of the thermal water and surrounding seawater is usually between 5.4 and 6.2, below the typical seawater pH of 8.5 (Carvalho et al. 2011). Additionally, pH fluctuations occur during the day as result of low and high tides, and sea conditions. This bacterium shows a significant plasticity being able to grow and produce prodigiosin at different pH values (Pereira and de Carvalho 2024a,b), probably as result of fluctuating conditions found at the sampling site.

The *S. rubideae* strain grows well in Marine Broth (MB; Pereira and de Carvalho 2024a, b). This medium appears inherently as a suspension since several of its constituents do not dissolve even after sterilization (Pronadisa 2020). In shake flasks, pH control is often achieved by buffers, but the species in the buffering system should not interfere with the media composition (Ferguson et al. 1980). Since MB is an enriched salt medium, containing ca. double the mineral content of sea water (Zobell 1941), the impact of the addition of phosphate buffer on medium composition, biomass produced and product concentration, was assessed. Several other buffers were applied as possible alternatives, and their impact on the bioprocess was studied and compared.

### Effect of Phosphate Buffer on Medium Composition, Bacterial Growth and Prodigiosin Production

The medium most suitable to grow the *S. rubidaea* strain and to enhance the production of prodigiosin was found previously to be MB supplemented with sodium glutamate, meat peptone and iron(III) sulfate (referred to as MB + S) (Pereira and de Carvalho 2024a, b).

**Fig. 1:**
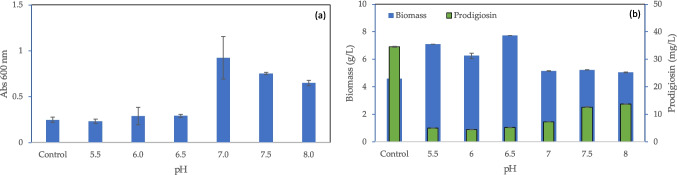
Influence of pH of cultivation medium on its turbidity without cells (**a**), and on biomass and prodigiosin production (**b**). Final pH was achieved by addition of phosphate buffer to MB + S. Control refers to MB + S, without buffer added, which has a pH of 6.3

To study which pH value promotes prodigiosin production, phosphate buffer solutions containing Na_2_HPO_4_/KH_2_PO_4_, at 45 mM and pH values between 5.5 and 8.0, were used to prepare the MB, and compared to MB + S. The effect of phosphate on the media components was assessed initially without cells. It was observed that, prior to heat sterilization, MB containing phosphate buffer with pH values ranging from 7.0 to 8.0 presented a higher turbidity when compared to the control, as shown by optical density measurements at 600 nm (Fig. 1a). This is an indication of precipitation of MB components at higher pH.

**Fig. 2:**
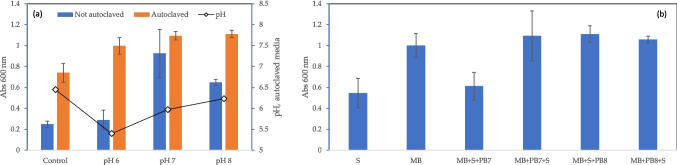
Effect of sterilization of the cultivation medium containing phosphate buffer on turbidity when all components are autoclaved together (**a**) or separately (**b**). On **b**, the order of addition of the different components is reflected in the name of each condition tested. Control refers to MB + S, without buffer added, which has a pH of 6.3

To assess the effects of the heat and pressure used during sterilization on each medium, they were sterilized in an autoclave at 121 °C and 1 bar (relative pressure). Measurements at 600 nm showed a 1.2- to 4.0-fold increase in turbidity of the buffered media after sterilization when compared to the corresponding medium before sterilization (Fig. 2a). Curiously, an increase in turbidity values after sterilization was observed with increasing pH values of the phosphate buffered medium. It was also observed that the turbidity values for the medium supplemented with phosphate buffer at pH 7.0 and 8.0 differed only in 1.5%. Additionally, pH measurements carried out after sterilization of MB + S indicated a pH below the value set before sterilization. This was the result of the precipitation of chemical species.

To determine which ingredient in MB + S was responsible for precipitation, the supplements sodium glutamate, meat peptone and ion(III) sulfate (all designed by “S”), the MB, and the phosphate buffer species (represented by PB7 and PB8 for final pH values of the cultivation medium of 7 and 8, respectively) were autoclaved separately, and added to each other in different order. When PB7 and PB8 were added to MB prior to S, a 9% increase and 1% decrease in turbidity were observed, respectively, when compared to MB (Fig. 2b). When compared to the case where PB was added at the end, a 1.2-fold increase was observed at pH 8 and a 0.7-fold decrease was observed at pH 7. This is a clear indication that phosphate buffer is inducing the precipitation of compounds present in MB.

The influence of media precipitation on *S. rubidaea* growth and prodigiosin production was also assessed. By comparison with the control MB + S, the amount of prodigiosin produced decreased between 2.5- and 6.8-fold when phosphate buffer was added, being the highest production observed at pH 8.0 (Fig. 1b). In fact, prodigiosin production increased with pH value but never reached the value observed for MB + S. At pH values lower than 7.0, the amount of DCW increased ca. 50% in comparison to the control culture, but at pH 7.0 to 8.0, the amount of DCW present was similar to that observed in the control (Fig. 1b).

### Effect of Non-phosphate Buffers on Bacterial Growth and Prodigiosin Production

**Fig. 3:**
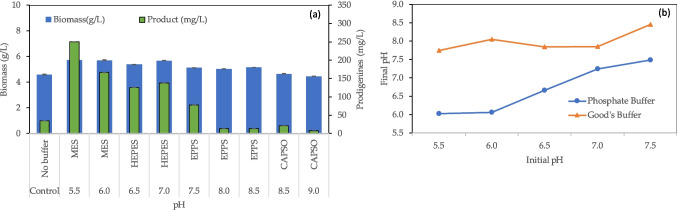
Influence of Good’s buffers on *S. rubidaea* growth: **a** biomass and prodigiosin production in MB + S at different pH values; **b** pH value measured at 24 h of cultivation when phosphate buffer and Good’s buffers were used. Control refers to MB + S, without buffer added, which has a pH of 6.3

Precipitate formation suggests that part of MB compounds became unavailable for *S. rubidaea* consumption, thus affecting cell metabolism and maintenance. To address this issue, several Good’s buffers were selected to control pH in shake flasks, as an alternative to phosphate buffer. CAPSO, EPPS, HEPES and MES were chosen, and used within their buffering range (Table 1). When compared to the control medium, these buffers enhanced biomass yield 1.13-fold (Fig. 3a). However, biomass production was 10 to 44% higher when phosphate buffer was used at pH values lower than 7, in comparison to the use of MES and HEPES (Figs. 1b and 3a). At pH 8.0 and above, when EPPS and CAPSO were used as buffers, prodigiosin concentration decreased at least to a third of that obtained in non-buffered MB + S (Fig. 3a). On the contrary, prodigiosin production had a significant increase at pH values below 7.0, reaching a 49.5-fold increase when MES was used to buffer the MB + S to pH 5.5 (Figs. 1b and 3a). In this case, a prodigiosin concentration of 249.8 mg/L was attained. Looking at the pH values of the growth medium after 24 h of cultivation, Good’s buffers showed a lower buffering effect than phosphate, the final medium pH being close to 8 (Fig. 2b). *S. marcescens* cells have been shown to have a strong buffering capacity and, regardless of the initial pH of the cultivation media, the final pH is usually between 7.2 and 8.0 (Williams and Qadri 1980). Using Good’s buffers, a similar behaviour was observed with *S. rubidaea* in the present study (Fig. 3b).

**Fig. 4:**
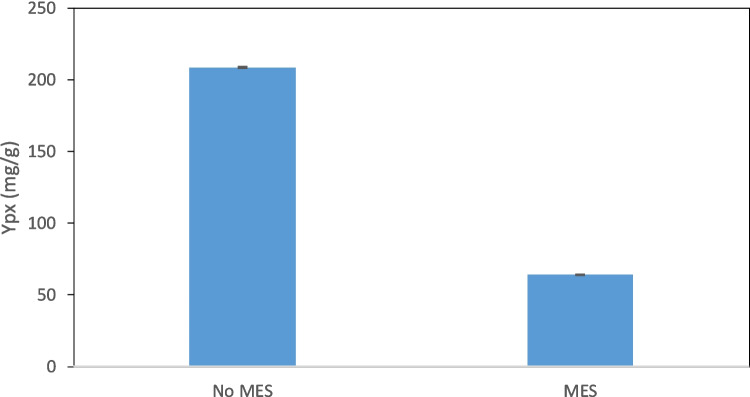
Influence of MES buffer on product yield (mg prodision/g cell dry weight). Bacterial growth and prodigiosin production were carried out in batch in a 2-L bioreactor containing MB + S medium, at 30 °C and pH 5.5, aerated at 1 vvm, with a 40% saturation of dissolved oxygen, and stirred at 450–600 rpm (controlled by aeration)

To assess if the higher production of prodigiosin observed in Fig. 3 was the result of MES buffer or only of the pH value of the medium, the bioprocess was carried out in a 2-L bioreactor and the pH was monitored and controlled in real time. According to the results presented in Fig. 3, the culture presenting the highest prodigiosin concentration was grown at pH 5.5 using MES as buffer. In one of the tested conditions in bioreactor, the cultivation medium contained MB + S and MES buffer, whilst in the other only MB + S was present. The pH was maintained at the set point (pH 5.5) in both conditions by automatic addition of 3N H_2_SO_4_ and 2N NaOH. Contrary to what was observed in shake flask (Fig. 3a), the presence of MES in the culture medium exerted an inhibitory effect on *S. rubidaea* cells, in particular in prodigiosin production, decreasing 3.0-fold the product to biomass yield in the bioreactor (Fig. 4). Although the difference between DCW concentration was 1.4-fold, the production of prodigiosin was 4.7-fold higher when MES was absent, resulting in a product to biomass yield of ca. 200 mg/g. MES is a zwitterionic buffer containing 2-(*N*-morpholino)ethanesulfonic acid with a pKa of 6.1 which has been found to be a competitive inhibitor of metallo-β-lactamases (Fitzgerald et al. 1998), an inhibitor of mycosubtilin production which is a lipopeptide antifungal biosurfactant (Guez et al. 2021), and to interfere with cellular phenotype (Vilariño et al. 1997). A metallo-β-lactamase is involved in the biosynthesis of prodigiosin in *Serratia* sp. (Gristwood et al. 2011) and it was shown that MES can be a competitive inhibitor of the metallo-β-lactamases by binding to the active site of the enzyme through the oxygens of the sulfonic acid group and the nitrogen of the morpholino ring (Fitzgerald et al. 1998). This could have been enhanced in the bioreactor by better mixing and the maintenance of pH at 5.5, when compared to the shaken flasks. In the flasks, since no control of pH was promoted besides the presence of the buffer, a pH of 7.8 was measured at 24 h when the cells were harvested.

### pH 7.0 Buffer Selection

The large majority of enzymes show maximum catalytic activity at pH values close to neutrality (Lund et al. 2020). As shown in the previous point, pH 5.5 could enhance the catalytic activity of the enzymes involved in the metabolic pathway of prodigiosin in *S. rubidaea* (Fig. 3a). However, using culture conditions where pH values are acidic is known to cause corrosion in the constructing materials of bioreactor vessels, especially in 316-L stainless steel (Nik Masdek et al. 2021). From an industrial perspective, rapid degradation of the materials can be the difference between the implementation or not of a bioprocess (Kip and Van Veen 2015; de Carvalho 2018; Procópio 2019).

**Fig. 5:**
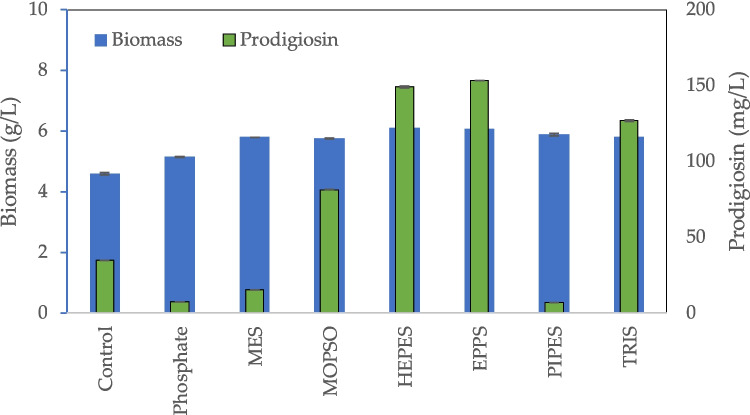
Influence of Good’s buffers, used for buffering the cultivation medium to pH 7.0, on *S. rubidaea* growth and prodigiosin production. Control = MB + S without any buffer added. The data is organized by chemical nature of the buffer

In the present experiment, the objective was to evaluate biomass and prodigiosin production in culture flasks, using pH 7.0 buffered media by phosphate and other species. Using prodigiosin production as model system, comparison between EPPS, HEPES, MES, MOPSO, PIPES and TRIS buffers with phosphate buffer was carried out. When necessary, the initial pH was adjusted to pH 7.0 using a 2 M sodium carbonate solution since the carbonate system regulates the pH of sea water (Millero 2000). The biomass concentration increased on average 28% in relation to the biomass concentration produced in the control culture, and 15% in comparison to MB + S buffered with phosphate (Fig. 5). The highest prodigiosin concentrations were observed in the presence of EPPS, HEPES and TRIS, being its concentration of 153.1, 148.8 and 126.8 mg/L, respectively (Fig. 5). MES and MOPSO are *N*-substituted aminosulfonic acids containing a morpholinic ring and should not form complexes with the main metal ions in the medium, whilst EPPS, HEPES and PIPES contain a piperazinic ring and should present a low ability to complex metal ions. On the contrary, TRIS is a primary amine which could be reactive, besides being able to penetrate biological membranes and to form complexes with several metal ions (Ferreira et al. 2015). The high prodigiosin concentration observed in the presence of TRIS indicates that other phenomenon should occur to counteract the expected inhibition in biological activity. When compared to the results shown in Fig. 3a, it is possible to observe that, for the same buffer, similar biomass production was observed for the different pH values but prodigiosin production could be largely affected (Fig. 5). For example, prodigiosin production in EPPS buffered media decreased from 153.1 mg/L at pH 7.0 (Fig. 5) to 13.8 and 13.7 at pH 8.0 and 8.5, respectively. Since at 30 °C the pKa value of the zwitterionic buffer EPPS is 7.89, the results indicate that additional negative charges in the buffer molecules occurring at pH values higher than the pKa may hamper prodigiosin production. Additionally, PIPES has also been found to be a competitive inhibitor of metallo-β-lactamases, although less potent than MES (Fitzgerald et al. 1998). This may explain the very low concentration of prodigiosin attained in its presence.

### Multivariate Analysis of pH Effect on the Bacterial Membrane

As it was shown in the previous sections, different buffers and pH values influenced prodigiosin production in different ways. To assess the behaviour of *S. rubidaea* cells during production, a lipidomics-based analysis was performed. It was previously shown in our group that, by studying the FA composition of the cellular membrane, it is possible to assess how bacteria such as *Rhodococcus erythropolis* may adapt to the substrate used as carbon source (de Carvalho et al. 2005; Rodrigues and de Carvalho 2019), to temperature, pH and presence of metal ions during growth (de Carvalho 2012), and to the surface to which they attach (Rodrigues and de Carvalho 2015).

In this study, a total of 57 fermentations were carried out as presented in the previous sections: 15 with phosphate buffer, 6 with CAPSO, 12 with EPPS, 6 with HEPES, 9 with MES and 3 with each of MOPSO, PIPES and TRIS. The FA composition of the cells in each fermentation was determined by gas chromatography.

**Fig. 6:**
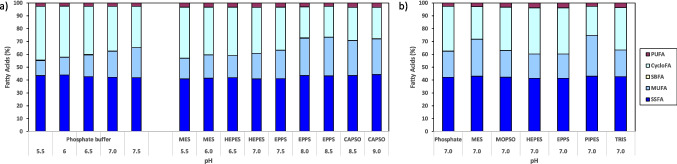
Effect of pH and buffer species on the lipid profile of the membrane of *S. rubidaea* cells. Fatty acid profiles of cells grown for 24 h with different buffer species at different pH values (**a**), and at pH 7.0 (**b**). Abbreviations: SSFA, saturated straight FA; MUFA, monounsaturated FA; SBFA, saturated methyl branched FA; CycloFA, saturated cyclopropyl branched FA; and PUFA, polyunsaturated FA

The FA profiles of the *S. rubidaea* cells grown with different buffer species and pH values showed that the adaptation of these cells is mainly related to the pH value and less to the type of buffer (Fig. 6a). A similar lipid profile of the cells grown at the same pH value was observed when different buffer species were used, whilst profile differences were observed between the cells grown in the presence of the same buffer but at different pH values. As the pH of the medium increased from 5.5 to 7.5, there was an increase in monounsaturated fatty acids (MUFA) concentration of 11.5% and 6.0%, and a decrease in saturated cyclopropyl branched fatty acids (CycloFA) of 9.6% and 6.4%, when the cells grew, respectively, in PO_4_ and in the Good’s buffers. These alterations should result in an increased fluidity of the cellular membrane with increasing pH values. Under acidic stress conditions, the *S. rubidaea* cells developed a membrane-shielding effect. Cyclopropane FAs reduce the fluidity of the cellular membrane but they are, in general, more ordered than the corresponding unsaturated chains, thus contributing to the stability of the membrane while decreasing the membrane permeability to protons (Gianotti et al. 2009; de Carvalho and Caramujo 2018; Lund et al. 2020). With increasing pH values, the cells increased the content in MUFA while decreasing the content in CycloFA (Fig. 6a). MUFAs are produced to maintain membrane fluidity since their synthesis is more energy-efficient in comparison with CycloFA (Zhang and Rock 2008). In the presence of PO_4_, *S. rubidaea* cells had more difficulty growing in MB + S then in media with Good’s buffers (Fig. 5), probably due to a larger energy expenditure required to make the necessary alterations in the membrane lipid content (Fig. 6).

When the initial pH of the medium was above 7.5, an increase in saturated straight fatty acids (SSFA) and MUFA concentrations of 2.2% and 7.9%, and a decrease in CycloFA of 9.4%, respectively, were observed (Fig. 6a). Interestingly, above pH 8.0 the lipid profile remained partially unchanged even in the presence of different buffer species (Fig. 6a). Since the seawater is on average at pH 8–8.5, this strain seems to be relatively well adapted to grow under marine conditions without the need to carry out significant changes in the cellular membrane above pH 7.5.

When the lipid composition of the cellular membrane of *S. rubidaea* cells grown at pH 7.0 in media buffered by different chemical species was studied, it was observed that the cells presented similar FA composition with the exception of medium buffered with PIPES and MES (Fig. 6b). In the presence of the latter buffers, the cells increased the content of MUFA while decreasing the content of CycloFA. These two buffers are chemically related, PIPES is an ethanesulfonic acid buffer whilst MES is (2-(*N*-morpholino)ethanesulfonic acid), which could influence the response of the cells. However, HEPES and EPPS also contain a sulfonic acid moiety but the cells did not increase the amount of MUFA in their presence.

To establish the correlation between pH values, buffer composition, lipid membrane composition and prodigiosin production, a multivariate analysis was necessary. Principle Component Analysis (PCA) is an unsupervised statistical method that aims at transforming a number of possibly correlated variables into a smaller number of uncorrelated orthogonal variables (principal components, PCs) that explain the variability of the larger set of variables (Jongman et al. 1995).

**Fig. 7:**
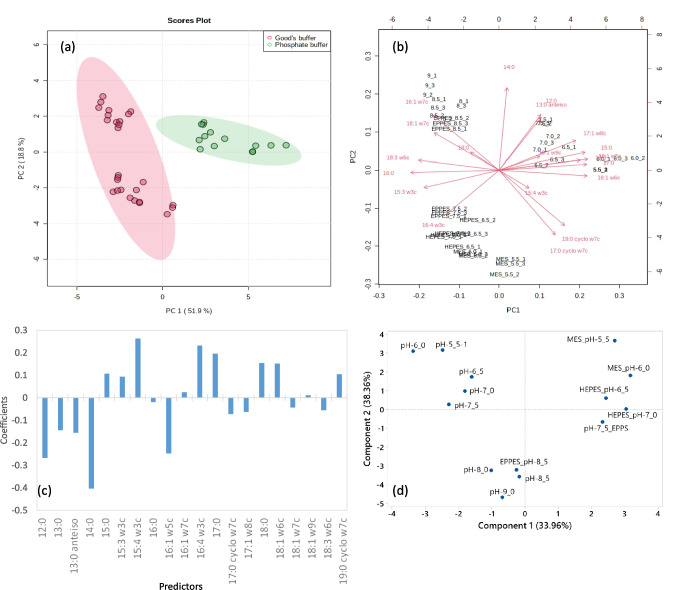
Principal component (PCA) and partial least squares (PLS) analyses. Score plot for PCA (**a**) and PLS (**d**) represented for the selected principal components (PCs), with explained variance shown in brackets. Loadings of the fatty acid in the plane created by the first to axes resulting from the PCA (**b**). Coefficients of the PLS model relating fatty acid composition of the cells grown in the presence of the various buffers tested and prodigiosin production (**c**)

In the present study, PCA could cluster data obtained with phosphate buffered media and with the Good’s buffers used: PC1 which explains 51.9% of the variance of the data could separate the data into 2 large groups (Fig. 7a). On the left-hand side of the axis of PC1 are data corresponding to cells grown in the presence of Good’s buffers, whilst on the right-hand side is the cluster corresponding to data concerning cells grown with phosphate buffered media. The first two PCs account for 70.7% of the variance of the data (Fig. 7a).

The relative contribution of each original variable to the PCs (loadings) is directly proportional to the length of the variable vector on the PCs space (Fig. 7b). The FAs 16:1 ω7c and 18:1 ω7c contributed more to the cells grown on Good’s buffers at higher pH values, whilst 16:4 w3c FA contributed more for distinguishing the cells grown on Good’s buffers at lower pH values. Cells grown on phosphate buffered media had higher vectors corresponding to the FAs 15:0, 17:0, 16:1 ω5c, 17:1 ω8c and 18:1 ω6c (Fig. 7b). *S. rubideae* cells presented larger content of 16:0 and 17:0 cyclo ω7c, followed by 18:1 ω7c and 19:0 cyclo ω7c (data not shown). The data separation was thus mainly achieved by PCA by FAs present in less than 5% (each) of the total FA content.

Partial Least Squares (PLS) is another statistical tool that allows the development of predictive models when the number of variables or factors in a system is large and they are highly collinear (Geladi and Kowalshi 1986; Denham 1995). PLS maximizes the covariance between the original variables while reducing the number of calculated predictive factors. In the present study, PLS was carried out using a matrix containing the FA composition of the *S. rubidaea* cells in the 57 fermentations and, as response array, the respective concentration of prodigiosin attained. The coefficients of the PLS were highest in modulus for the FAs 14:0 (− 0.40), followed by the 12:0 (− 0.27), 15:4 ω3c (+ 0.26), 16:1 ω5c (− 0.25) and 16:4 ω3c (+ 0.23) FAs (Fig. 7c). Short SSFA and long MUFA were inversely correlated with prodigiosin production, whilst long SSFA and PUFA were directly correlated with prodigiosin concentration.

In fact, when the scores of the model are represented in the plane formed by components 1 and 2, which represent 72.3% of the variance, three clusters are shown (Fig. 7d). Component 1 separates the data clusters according to the presence of phosphate buffer (left-hand side of Fig. 7d) and Good’s buffers (right-hand side). Component 2 separates the data according to the pH value of the buffer used: the bottom cluster is formed by data corresponding to the cells grown in pH higher than 7.5, whilst the top of the chart shows data corresponding to cells grown in pH values equal or below 7.5. When comparing the PCA and PLS score plots of the model (Fig. 7a, d), it is possible to conclude that both multivariate analyses show that *S. rubidaea* cells changed their FA composition in response to the buffer used and the respective pH value in the cultivation media, and that the type of buffer also influenced prodigiosin production.

### Multivariate Analysis of Buffer Type on Bacterial Membrane

**Fig. 8:**
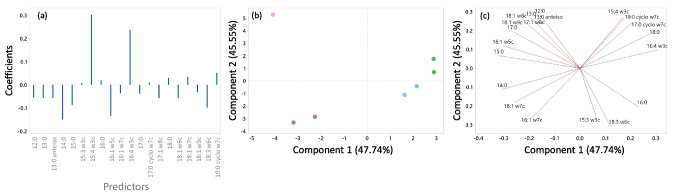
Partial least squares analysis. **a** Regression coefficients; score (**b**) and loading (**c**) plots of PLS using prodigiosin concentration as response and fatty acids as variables. HEPES and EPPS (  ), TRIS and MOPSO (  ), MES and PIPES (  ) and phosphate (  )

As mentioned in the previous section, the lipid profile of *S. rubidaea* cells grown in media with pH 7.0 was changed only when the cells grew in medium buffered by PIPES and MES (Fig. 6b), resulting in lower production of prodigiosin (Fig. 5). To assess the influence of the type of buffer selected to maintain the medium pH at 7.0 on cell behaviour, multivariate analysis was also performed to determine which FA were changed. A PLS model, obtained from the matrix containing the composition of FA of the lipids extracted from *S. rubidaea* cells grown at pH 7 in the Erlenmeyer flasks, and having as a response array the corresponding prodigiosin concentration, showed coefficients with higher values for the FA 15:4 w3c (0.311) and 16:4 w3c (0.256) (Fig. 8a).

Looking into the scores plot (Fig. 8b), where components 1 and 2 of the model represent 93.3% of the variance, three clusters could be found. Component 1 separates the data according to the buffer type. The two clusters on the left-hand side are respectively phosphate (top) and buffers with molecules without an available hydroxyl group (bottom). The third cluster on the right-hand side corresponds to different molecules which have more than one available hydroxyl group. Component 2 separates the data according to the similarity between molecules. HEPES and EPPS have the same base molecule, TRIS and MOPSO have a secondary or a tertiary carbon present, MES and PIPES do not have any hydroxyl groups, and phosphate is the most different being the only molecule, between the selected buffers, with a phosphorus group. The relative contribution of each FA to the principal components (loadings) is proportional to the projection of its vector: prodigiosin-producing cells when grown with EPPS or HEPES have higher contribution of 16:4 w3c; in TRIS and MOPSO, a larger contribution of 16:0; with MES and PIPES, the more prominent FA are MUFA with 16 and 18 carbon atoms; and on phosphate, larger contribution of 17:0 could be observed (Fig. 8c).

## Conclusions

From the surface to the deep ocean, temperature and pH gradients are present and their values are hardly constant. This variability affects the characteristics of the proteins produced by marine microorganisms, namely the enzyme rates and stability. This may affect the productivity of interesting products. In this study, to test the plasticity of this *S. rubidaea*, an extremophile, and its behaviour regarding pH, prodigiosin production was used as model system. Supplementation of MB + S at different pH values, ranging from 5.5 to 7.5 and maintained by using phosphate buffer, was carried out, and it was shown that prodigiosin production was higher at neutral to basic pH values than at acidic conditions. However, in comparison with unbuffered MB + S, the amount of prodigiosin produced was 6.8-fold lower. It was noticed that phosphate interfered with the other components of the medium causing their precipitation, being this quite notorious after medium sterilization, even prior to inoculation.

During the search for an alternative buffer system, different Good’s buffers were tested, within their respective buffering region. Although Good’s buffers showed a lesser buffering effect than phosphate, prodigiosin production at pH values below 7.0 reached a 5.2-fold increase in comparison with control cells grown in MB + S without buffer.

To guarantee the possible implementation of this bioprocess industrially, EPPS, HEPES, MES, MOPSO, PIPES, TRIS and phosphate buffers were used to maintain the MB + S medium at pH 7.0. The highest concentration of prodigiosin attained was 153.1 mg/L in the presence of EPPS. Looking into the chemical structure of the two Good’s buffers used when prodigiosin concentration was highest, MES and EPPS, it suggests that the difference in production attained with these buffers was linked to their negative charges, due to the selection of a pH value higher than their pKa.

The results from the multivariate analysis relating the lipid composition of the cells and the growth/production conditions showed that it was the pH value of the medium, and not the buffer species, that led to the largest variations on the lipid profile of the cellular membrane, and consequentially to the cell growth and product production.

Using prodigiosin production as model system to evaluate the effect of pH, it was shown that the different pH values influenced the production rate of prodigiosin which varied from 10.4 mg/(L.h) to 0.2 mg/(L.h), being mostly influenced by pH values as they change from acidic to basic. However, production occurred at every pH value tested, showing the adaptive capacity of this *S. rubidaea* strain to survive and thrive at environments whose pH values may rapidly change. Based on our previous work with this strain [16], we may conclude that the strain has the characteristics of a polyextremophyle since it can survive and adapt to a myriad of extreme abiotic conditions, namely: temperature between 15 and 62 °C; salt concentration ranging from 20 to 80 g/L; and pH between 5.5 and 9.0. The capabilities of this marine *S. rubidaea* makes it a good candidate, and in particular its high tolerant enzymes, for application in industrially relevant bioprocesses.

## Data Availability

Data will be made available on request.
